# Noncanonical bactericidal activity of teleost type I interferon is conferred by a membrane-targeting C-terminal peptide

**DOI:** 10.1371/journal.ppat.1014419

**Published:** 2026-07-28

**Authors:** Han Zhang, Ying Wu, Xiaoyuan Zheng, Ziyu Wang, Chen Zhang, Zhenjie Cao, Jingqun Ao, Yongcan Zhou, Yun Sun

**Affiliations:** 1 Sanya Institute of Breeding and Multiplication, School of Marine Biology and Fisheries, Collaborative Innovation Center of Marine Science and Technology, Hainan University, Hainan, China; 2 Engineering Research Center of Hainan Province for Blue Carbon and Coastal Wetland Conservation and Restoration, Hainan University, Hainan, China; 3 International Joint Research Center of Hainan Province for Blue Carbon and Coastal Wetland, Hainan University, Hainan, China; 4 College of Marine Sciences, College of Life Sciences, Fujian Agriculture and Forestry University, Fuzhou, China; Universidade do Porto Instituto de Investigacao e Inovacao em Saude, PORTUGAL

## Abstract

Type I interferons (IFNs) are indispensable antiviral cytokines in nonspecific immunity, yet they play dual roles in bacterial infections in mammals. Recent studies have revealed a subset of strongly cationic type I IFNs possessing potent antimicrobial properties across nonmammalian vertebrates. In this study, we identified a type I IFN gene, *Ca*IFNi, from *Cromileptes altivelis* that is characterized by a unique triple-disulfide bond architecture. In *Vibrio harveyi*-challenged models, overexpression of *Ca*IFNi potentiated bacterial clearance capacity in tissues, whereas its knockdown exacerbated bacterial colonization, highlighting its ability to protect the host against bacterial infection *in vivo*. *In vitro* assays further confirmed that *Ca*IFNi directly binds to and kills both gram-negative (G^-^) and gram-positive (G^+^) bacteria, which first revealed the antibacterial function of new subgroup IFNi within teleost type I IFNs. Furthermore, the α-helical peptide *Ca*IFNi-18 derived from *Ca*IFNi was identified as a novel antimicrobial peptide (AMP) that has broad-spectrum antibacterial efficacy against G^-^ and G^+^ bacteria and membrane-targeting ability. Further mechanistic studies revealed that *Ca*IFNi has bactericidal effects on both G^-^ and G^+^ bacteria through membrane depolarization and disruption, alteration of the bacterial ultrastructure, and *in vitro* binding to genomic DNA. In addition, *Ca*IFNi-18 also has significant *in vivo* therapeutic efficacy against bacterial infection, highlighting its great potential as an antibacterial agent. Encouragingly, the loss of antibacterial activity in the truncation mutant (r*Ca*IFNiΔ148-165) lacking the *Ca*IFNi-18 segment suggests that this region is essential for the bactericidal function of the full-length protein and likely acts as its core domain. Further computational simulations revealed that the deletion of the *Ca*IFNi-18 region attenuated the interaction between the protein and the bacterial membrane. These findings not only expand the functional scope of type I IFNs beyond their canonical antiviral role but also identify their derivative *Ca*IFNi-18 as both a promising antimicrobial candidate and the essential bactericidal domain of *Ca*IFNi, thereby offering novel therapeutic strategies against bacterial infections in the aquaculture industry and beyond.

## Author summary

Interferons are classically recognized as key immune cytokines that defend hosts against viral infections. However, the pathogen-rich aquatic environment has driven teleost fish to evolve uniquely versatile immune defenses. In this study, we characterized a specific type I interferon (*Ca*IFNi) from the humpback grouper (*Cromileptes altivelis*) and uncovered its surprising ability to directly kill both Gram-negative and Gram-positive bacteria. We demonstrate that this noncanonical antibacterial function is governed by a small, positively charged segment located at the protein’s C-terminus, designated *Ca*IFNi-18. This short peptide functions as a potent antimicrobial agent by directly targeting and disrupting bacterial membranes. Notably, deleting this segment completely abolishes the full-length interferon’s capacity to bind to and kill bacteria. Our findings reveal that a classic antiviral molecule harbors a critical domain that mediates both direct bactericidal activity and robust therapeutic protection against bacterial infections, which uncovers a new dimension of innate immunity in teleosts and highlights its potential for anti-infective drug design.

## Introduction

Interferons (IFNs), classified among class II alpha-helical cytokines, serve as indispensable components of both adaptive and innate immune responses [1,2]. IFNs are categorized into four types: IFN-I, IFN-II, IFN-III, and IFN-IV [3]. In teleosts, type I, II, and IV IFNs have been identified [4]. Type I IFNs are highly diversified across teleosts and can be divided into two distinct groups: Group I, which are characterized by two cysteines and are further subdivided into Groups a, d, e, and h; and Group II, which contain four or six cysteines and are subdivided into Groups b, c, f, and i [5]. In contrast to the ubiquitous presence of Group I IFNs in all teleosts, Group II IFNs exhibit a more constrained distribution and are found only in specific lineages, such as cyprinids, salmonids and perciformes [5,6].

While type I IFNs in mammals are known primarily for their central role in antiviral defense, their functional repertoire in teleosts has undergone remarkable expansion, encompassing not only antiviral immunity but also direct antibacterial activity. As a case in point, IFNd in large yellow croaker can elicit an antiviral response by upregulating ISG expression and suppressing the replication of giant salamander iridovirus in the GS and LYCK cell lines [7]. Similarly, the recombinant protein On-IFNc efficiently reduces the ISKNV viral load in MFF-1 cells [8]. Beyond their canonical antiviral functions, accumulating evidence has demonstrated that teleost type I IFNs also exert considerable direct antibacterial effects. Akin to antimicrobial peptides (AMPs), the cationic recombinant protein rgcIFNφ1 from grass carp not only possesses broad-spectrum antimicrobial activity against both gram-negative (G^-^) and gram-positive (G^+^) bacteria *in vitro* but also confers potent protection in zebraﬁsh against *A*. *hydrophila* infection *in vivo* [9]. Furthermore, a recent report revealed that in Chinese sturgeon, the Group II IFN protein r*As*IFNf, which has a positive charge, also displays wide-spectrum bactericidal properties *in vitro* [10]. Thus, type I IFNs in teleosts function as direct bactericidal agents because of their strong positive charge—an AMP-like trait.

AMPs are small molecular polypeptides (typically 12–50 amino acids) that are ubiquitously found across natural organisms and act as key effector molecules of innate immunity [11]. Unlike conventional antibiotics that target specific biosynthetic pathways, their cationic and amphipathic nature facilitates interactions with anionic phospholipids of the bacterial membrane and in turn triggers bacterial membrane disruption, which endows them with broad-spectrum antimicrobial activity [12]. Furthermore, AMPs exert antimicrobial effects through diverse modes of action, such as inhibiting biofilm formation, blocking nucleic acid replication, tuning immune responses and impairing core metabolic functions in microbes [11,13]. These multifaceted mechanisms are conducive to alleviating resistance development in bacteria and make them promising candidates for addressing antibacterial infections [14].

Humpback grouper (*Cromileptes altivelis*), a high-value mariculture species in the South China Sea, sustains diverse aquaculture stressors, culminating in disease outbreaks and substantial economic losses in the aquaculture industry [15]. In particular, bacterial infections pose a severe threat to humpback grouper aquaculture, among which *V. harveyi* emerges as a highly virulent pathogen. To combat these infections, antibiotic use constitutes the prevailing strategy, yet its indiscriminate application has exacerbated bacterial resistance and environmental contamination [16]. Therefore, the identification and development of ecologically sustainable antimicrobial agents is critical. IFNi, a new subgroup of type I IFNs first identified in *Larimichthys crocea*, was previously assigned to the IFNc subtypes of group II IFNs in light of phylogenetic analysis and four highly conserved cysteines [17]. Nevertheless, a recent study revealed that compared with IFNc, IFNi possesses two additional cysteine residues, leading to the formation of three disulfide bonds, which distinguishes it from other type I IFNs [5]. Phylogenetically, IFNi forms a distinct evolutionary clade within teleost group II IFNs. To date, functional characterization of IFNi has been limited primarily to *L. crocea*, where its expression is robustly induced by *Aeromonas hydrophila* and poly I:C, resulting in potent antiviral activity *in vitro* [17]. However, its antimicrobial activity and modes of action in bacterial infections are still poorly defined.

In the present research, we identified a Group II type I IFN (*Ca*IFNi) in *C. altivelis* and revealed for the first time its direct antibacterial activity both *in vitro* and *in vivo*. Furthermore, the C-terminal-derived cationic peptide *Ca*IFNi-18 was identified as a novel, membrane-targeting AMP with broad-spectrum antibacterial activity and high biocompatibility. More importantly, by employing a truncation mutant (r*Ca*IFNiΔ148-165) coupled with computational simulations, we demonstrated that the *Ca*IFNi-18 peptide region is essential for the antibacterial activity and membrane interaction of the full-length protein, as its deletion abrogated bioactivity. Therefore, our work not only reveals a noncanonical antibacterial function for teleost type I IFNs but also precisely identifies its structural basis for functionality, highlighting *Ca*IFNi-18 as a promising therapeutic candidate and paving the way for novel antimicrobial strategies in aquaculture.

## Results

### Identification and bioinformatics analysis of *Ca*IFNi

**Fig 1:**
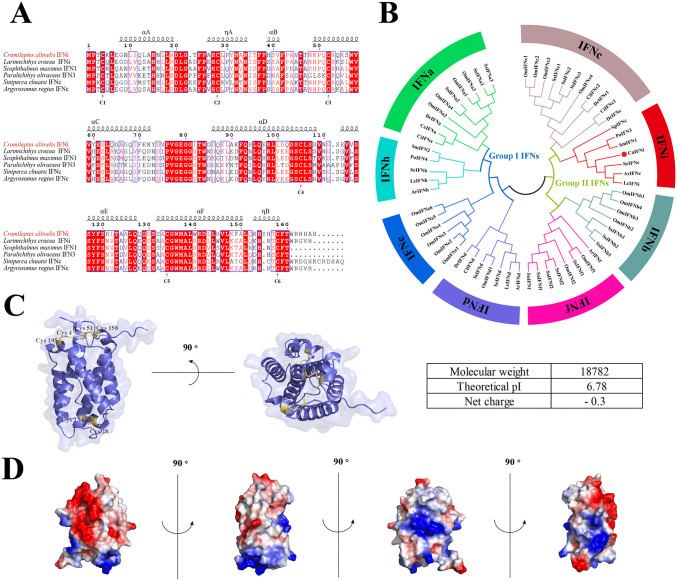
Sequence and structure analysis of *Ca*IFNi. **(A)** Multiple sequence alignment of the mature peptide sequence of *Ca*IFNi and fish homologs using the ESPript 3.0 server. Six conserved cysteine residues (C1–C6), which are essential for the formation of intramolecular disulfide bonds, are annotated. **(B)** Phylogenetic analysis of *Ca*IFNi and other teleost type I IFN amino acid sequences constructed by the neighbor-joining method using MEGA 9.0 software. The GenBank accession numbers of the amino acid sequences used for phylogenetic tree construction are provided in Table S2. **(C)** The 3D structure of *Ca*IFNi predicted by AlphaFold3. Disulfide bonds are highlighted in yellow. **(D)** Electrostatic surface potential mapping of *Ca*IFNi visualized via PyMOL. The hydrophobic, negatively charged and positively charged regions are colored white, red and blue, respectively.

The *Ca*IFNi gene, comprising a 567 bp ORF, encodes a 188-amino acid precursor with a putative signal peptide of 22 residues. As presented in Fig 1A and 1C, the mature peptide of *Ca*IFNi had a prototypical type I IFN tertiary structure characterized by six α-helices and contained six highly conserved cysteine residues that formed three pairs of disulfide bridges. To further elucidate the evolutionary relationship of *Ca*IFNi, a phylogenetic analysis based on multiple type I IFN subgroups in fish was performed, and we found that *Ca*IFNi clustered with *Lc*IFNi, *Ar*IFNc, *Sc*IFNc, *Sm*IFN1, *Po*IFN3 and *Sp*IFNc (Fig 1B), forming a novel clade designated previously as the IFNi group [5]. Computational prediction by ExPASy revealed that *Ca*IFNi possessed a molecular weight of 18.78 kDa and an isoelectric point (pI) of 6.78. Notably, electrostatic surface analysis revealed a localized accumulation of positive charge within the C-terminal region of *Ca*IFNi, in comparison with a mild negative overall net charge of − 0.3 (Fig 1D).

### Bacterial infection rapidly induces *Ca*IFNi expression

**Fig 2:**
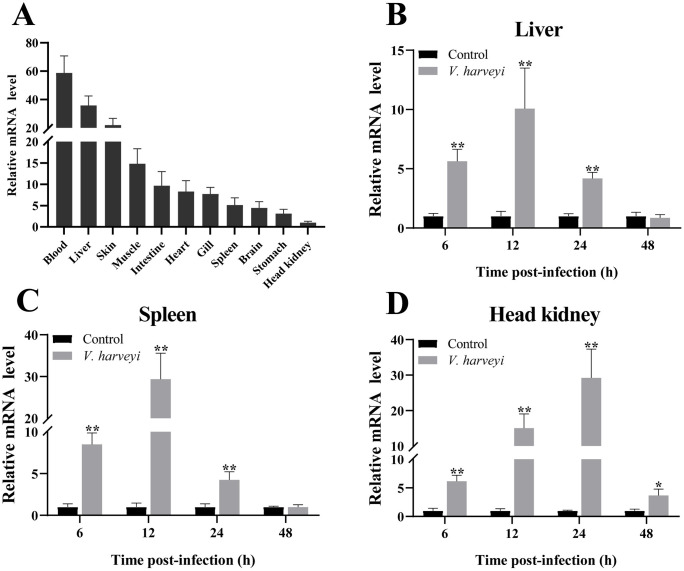
*Ca*IFNi is upregulated in humpback grouper after *V. harveyi* stimulation. **(A)** Tissue distribution of *Ca*IFNi among humpback grouper. **(B-D)** Expression profiles of *Ca*IFNi in the liver (B), spleen (C) and head kidney (D) of humpback grouper at 6, 12, 24, and 48 h post infection. The housekeeping gene *RPL13* was used as the internal control. Data are shown as the mean ± SD from one representative experiment (*n* = 3 biological replicates). Each replicate (*n*) represents a composite sample consisting of pooled tissues from five individual fish. (B–D) Statistical significance relative to the uninfected control at the corresponding time point was evaluated using an unpaired Student’s t test. *p < 0.05; **p < 0.01.

To determine the tissue distribution pattern of *Ca*IFNi, we carried out a qRT‒PCR analysis and revealed that it was ubiquitously expressed in all the collected tissues, with the highest mRNA levels detected in blood and the lowest in the head kidney (Fig 2A). Following *V. harveyi* stimulation, the expression of *Ca*IFNi increased significantly in the major immune tissues, peaking at 12 hpi in the liver (Fig 2B) and spleen (Fig 2C), whereas the maximum expression occurred in the head kidney at 24 hpi (Fig 2D). These results indicate that bacterial infection triggers rapid and robust transcriptional activation of *Ca*IFNi in immune-related tissues.

### *Ca*IFNi protects *C*. *altivelis* from *V. harveyi* infection

**Fig 3:**
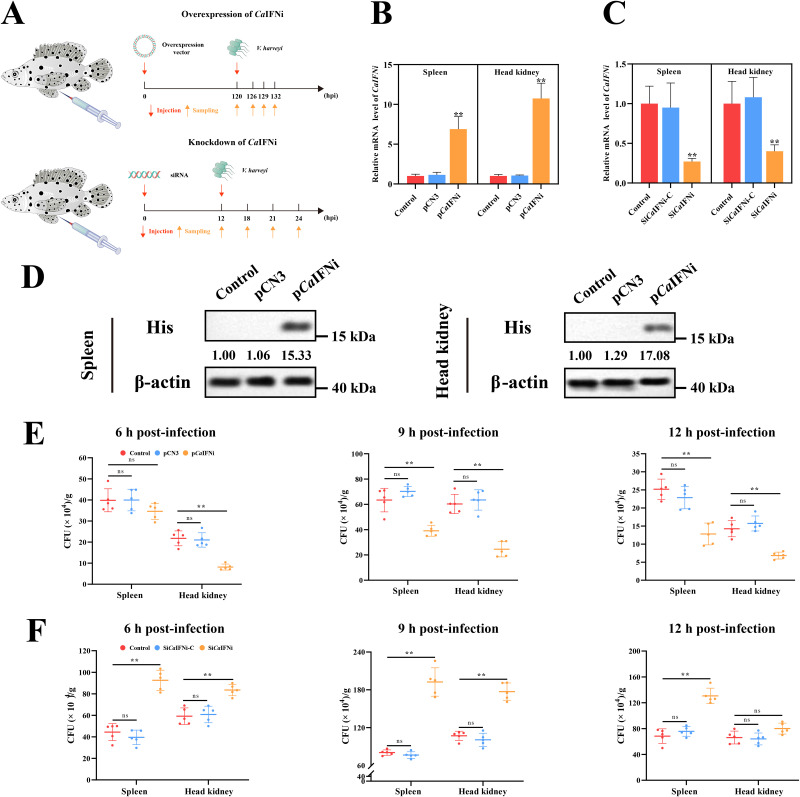
*Ca*IFNi protects humpback grouper from *V. harveyi* infection *in vivo.* **(A)** Schematic diagram of the *in vivo* experimental design. **(B-C)** The transcript level of *CaIFNi* in the immune tissues of groupers after injection with overexpression plasmids at 120 hpi (B) or siRNA at 12 hpi (C). *RPL13* was employed as the internal control. **(D)** Western blot analysis of *Ca*IFNi protein expression in the spleens and head kidneys of groupers injected with overexpression plasmids at 120 hpi using an anti-His antibody. β-actin was used as the control. **(E–F)** The groupers were injected i.p. with 5 × 10^4^ CFU of *V. harveyi* per fish. The bacterial burden in the spleen and head kidney was quantified at 6, 9, and 12 h post challenge under conditions of *Ca*IFNi overexpression (E) and knockdown (F). All the data are presented as the mean ± SD from one representative experiment (*n* = 3 biological replicates for B-C; *n* = 5 biological replicates for E-F). Statistical significance was evaluated using an unpaired Student’s t test for (B-C) and the Mann‒Whitney U test for (E-F). *p < 0.05; **p < 0.01.

To explore its resistance to bacterial infection, *Ca*IFNi was overexpressed in groupers via intramuscular injection with p*Ca*IFNi plasmids (Fig 3A). The successful overexpression of *Ca*IFNi at 5 dpi was confirmed at both the mRNA and protein levels in the spleen and head kidney via qRT‒PCR (Fig 3B) and Western blot analysis (Fig 3D), respectively. Subsequently, quantification of the bacterial burden in tissues revealed that the degree of bacterial colonization in the spleen and head kidney was significantly attenuated in the p*Ca*IFNi group compared with the control group at 9 and 12 hpi (Fig 3E). Notably, compared with those in the control group, the bacterial loads in the head kidney in the p*Ca*IFNi group were suppressed as early as 6 hpi, whereas those in the spleen were not significantly different (Fig 3E).

siRNA technology was used to interfere with the expression of *Ca*IFNi to further elucidate its role in antibacterial infection (Fig 3A). Similarly, qRT‒PCR analysis revealed a significant reduction in the mRNA expression of *Ca*IFNi among the examined tissues at 12 h after siRNA administration (Fig 3C). As illustrated in Fig 3F, following *V. harveyi* challenge, the bacterial colonies in the Si*Ca*IFNi-administration group were significantly greater in the spleen at 6, 9, and 12 hpi and in the head kidney at 6 and 9 hpi than those in the control group. These results suggest that *Ca*IFNi plays a pivotal role in resistance to bacterial infection *in vivo*.

### *Ca*IFNi binds to diverse bacteria and kills both gram-negative and gram-positive bacteria *in vitro*

**Fig 4:**
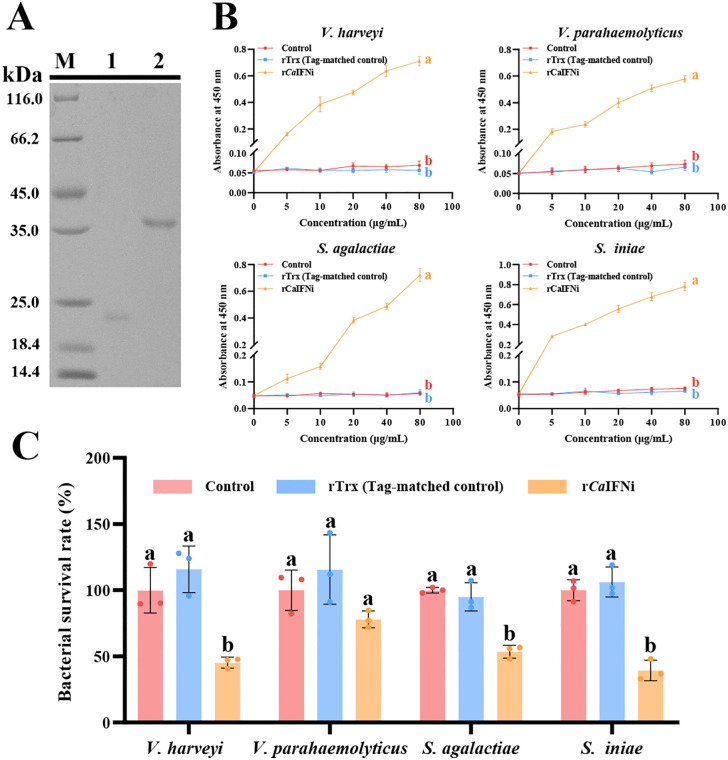
r*Ca*IFNi can directly bind to bacteria and exhibit bactericidal ability *in vitro.* **(A)** Analysis of recombinant *Ca*IFNi protein purified from *E. coli* by SDS‒PAGE. Lanes 1 and 2 represent the purified rTrx and r*Ca*IFNi proteins, respectively. **(B)** ELISA of r*Ca*IFNi binding to various aquatic bacterial pathogens. **(C)** Antibacterial effect of r*Ca*IFNi against bacterial pathogens determined via plate counting. PBS and rTrx treatments served as the control and tag-matched control, respectively. All the data are presented as the mean ± SD from one representative experiment (*n* = 3 independent samples). Statistical significance was evaluated using two-way ANOVA for (B) and one-way ANOVA for (C). Significant differences (*p* < 0.05) are denoted by different letters. For visual clarity in the ELISA curves (B), significance is marked exclusively at the highest concentration.

In addition to its anti-infection functions *in vivo*, we wondered whether *Ca*IFNi exhibits direct antibacterial activity *in vitro*. To this end, the recombinant protein r*Ca*IFNi, containing Trx and His tags, was expressed and purified to explore its functions *in vitro*. In light of the results of the SDS‒PAGE analysis presented in Fig 4A, the protein’s molecular weight (38.76 kDa) aligned with the predicted value. ELISA was performed to first investigate the binding activity of r*Ca*IFNi to various bacterial pathogens. As shown in Fig 4B, r*Ca*IFNi bound to both G^-^ bacteria (*V. harveyi* and *V. parahaemolyticus*) and G^+^ bacteria (*S. agalactiae* and *S. iniae*). A plate counting assay was subsequently conducted to further confirm its antibacterial activity *in vitro*. The results revealed that r*Ca*IFNi possessed significant antibacterial efficacy against *V. harveyi* (45.25%), *S. agalactiae* (53.57%) and *S. iniae* (39.34%), in contrast to the marginal bactericidal effects on *V. parahaemolyticus* (78.03%) (Fig 4C). These results reveal that r*Ca*IFNi binds to bacteria and has direct antimicrobial potency *in vitro*.

### *Ca*IFNi-18 is a novel cationic α-helical peptide localized in the C-terminal region of *Ca*IFNi

**Fig 5:**
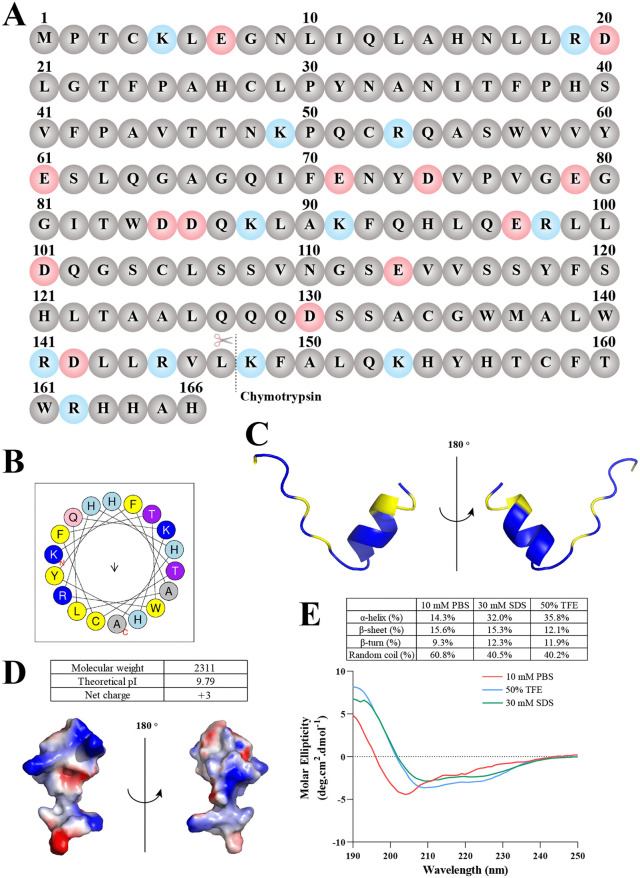
*Ca*IFNi-18 is a novel cationic α-helical peptide. **(A)** Predicted protease cleavage sites of CaIFNi-18 generated by ExPASy PeptideCutter **(B)** Helical wheel projection of *Ca*IFNi-18 generated by Heliquest. **(C)** The 3D structure of *Ca*IFNi-18 predicted by I-TASSER. PyMOL was utilized for visualization, resulting in a yellow, hydrophobic and blue, hydrophilic surface. **(D)** Electrostatic surface potential mapping of *Ca*IFNi-18 visualized via PyMOL. The hydrophobic, negatively charged and positively charged regions are colored white, red and blue, respectively. **(E)** Analysis of the *Ca*IFNi-18 secondary structure by CD spectroscopy.

Considering the aforementioned electrostatic surface of *Ca*IFNi (Fig 1D), we speculated that a cationic domain localized to its C-terminal region is likely responsible for the antibacterial activity of *Ca*IFNi. To validate this, the C-terminal short peptide *Ca*IFNi-18 was screened via AMPA and CAMPR4 servers, both of which predicted significant antibacterial potential, and then synthesized for follow-up assays. Additionally, our predictions revealed potential protease cleavage sites at positions 147 (Fig 5A), suggesting that the functional motif represented by *Ca*IFNi-18 may be naturally released *in vivo*. The helical wheel projection (Fig 5B) and electrostatic surface distribution (Fig 5D) revealed a cationic but nonamphiphilic feature in *Ca*IFNi-18, whereas the tertiary structure modeled by I-TASSER indicated that *Ca*IFNi-18 contained random coil regions and α-helix structures (Fig 5C). Furthermore, CD spectroscopy was utilized to further measure the secondary structures of *Ca*IFNi-18. As displayed in Fig 5E, *Ca*IFNi-18 predominantly adopted the random coil conformation in PBS, whereas its helix content increased markedly both in 30 mM SDS (simulating anionic prokaryotic membrane) and 50% TFE (modeling hydrophobic membrane), suggesting that *Ca*IFNi-18 might undergo a coil-to-helix transition upon interaction with the bacterial membrane.

### *Ca*IFNi-18 interacts with bacterial membranes by targeting LPS or LTA

**Fig 6:**
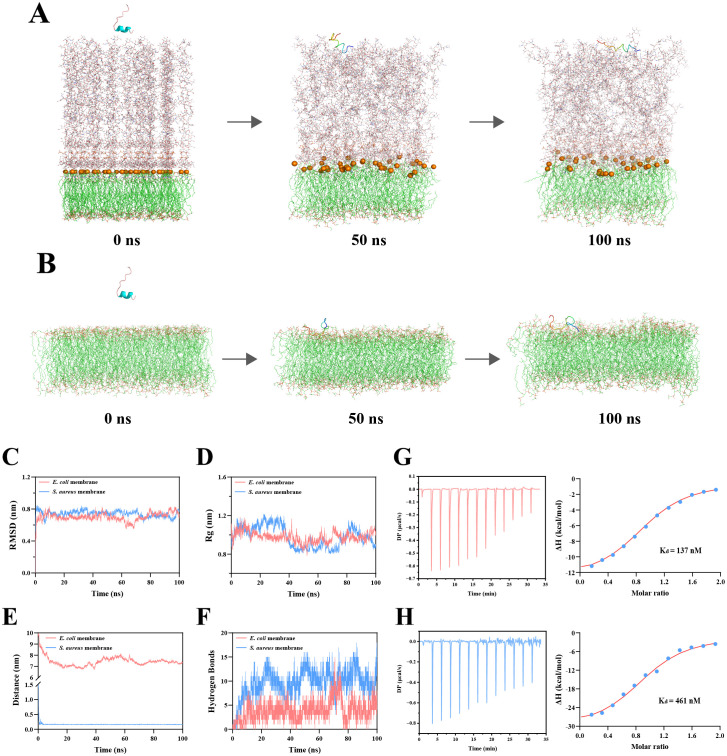
*Ca*IFNi-18 binds to bacterial membranes through its specific targeting of LPS or LTA. **(A-B)** MD simulation snapshots of the interaction between *Ca*IFNi-18 and the G^-^ outer membrane (A) or the G^+^ membrane (B) at 0, 50, and 100 ns. LPS is shown on the top leaflet of the G^-^ outer membrane, whereas the phosphorus atoms of lipid A are shown as tan spheres. **(C-D)** Variation in RMSD (C) and Rg (D) values for *Ca*IFNi-18 during MD simulations. **(E)** Distance between the COM of *Ca*IFNi-18 and the bacterial membrane during MD simulations. **(F)** Hydrogen bonds of peptide‒membrane interaction systems during MD simulations. **(G–H)** ITC assays for determining the binding of *Ca*IFNi-18 to LPS (G) or LTA (H). The right and left plots display the integrated heat measurements and corrected titration data, respectively.

Natural AMPs or antimicrobial cytokines, such as IL-26 and gcCXCL20a, are typically cationic and amphipathic, enabling electrostatic attraction to negatively charged bacterial membranes [18,19]. Most of them consistently appear to exert their functions starting from membrane interactions. Hence, we first employed all-atom MD simulations to characterize the binding of CaIFNi-18 to G^-^ (*E. coli*) and G^+^ (*S. aureus*) bacterial membrane systems. As clearly presented in Fig 6A and 6B, *Ca*IFNi-18 was found to associate with the membranes of *E. coli* and *S. aureus*, followed by partial embedding into the membrane systems. However, during the simulation processes, the α-helical conformation of *Ca*IFNi-18 destabilized and transitioned to a random coil, which aligned with the significant RMSD fluctuations of the peptide (Fig 6C). Consistently low Rg fluctuations manifested conformational compactness within the peptide (Fig 6D). Critically, decreasing COM distances (Fig 6E) coupled with increasing numbers of hydrogen bonds (Fig 6F) also signified peptide‒membrane complex formation. To further validate the theoretical predictions, we performed ITC to investigate the interactions between *Ca*IFNi-18 and bacterial membrane components (LPS and LTA). Similarly, the results revealed robust interactions between *Ca*IFNi-18 and LPS (K_d_ = 137 nM) or LTA (K_d_ = 461 nM) (Fig 6G–6H). Taken together, these results reveal that *Ca*IFNi-18 is capable of binding to both G^-^ and G^+^ bacterial membranes and targeting LPS and LTA.

### *Ca*IFNi-18 demonstrates potent broad-spectrum antimicrobial activity and excellent biocompatibility *in vitro*

**Fig 7:**
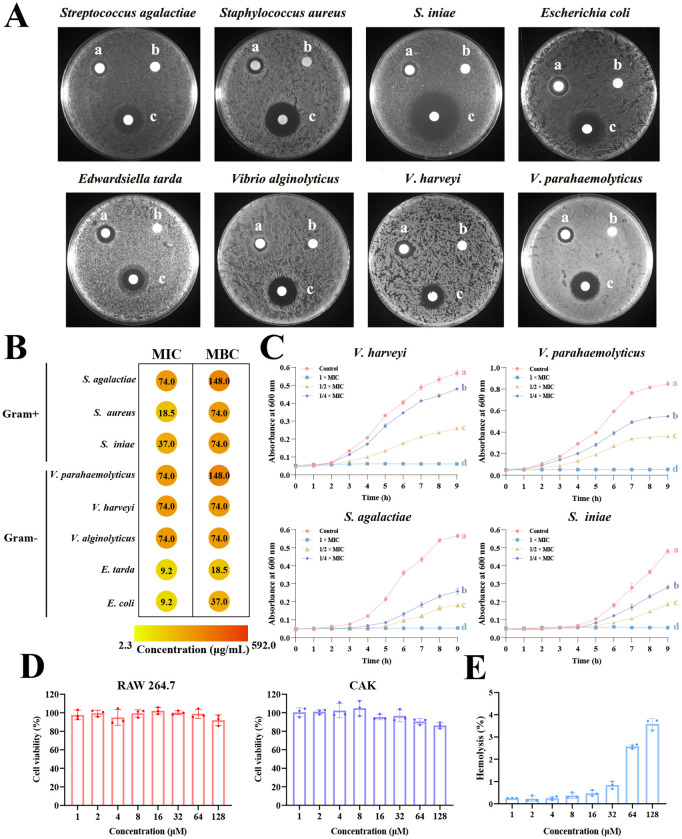
*Ca*IFNi-18 exhibits pronounced broad-spectrum antimicrobial efficacy coupled with notable biocompatibility *in vitro.* **(A)** Assessment of *Ca*IFNi-18 inhibitory zones against eight bacterial pathogens. Paper disks a, b, and c correspond to treatments with 1 mg/mL *Ca*IFNi-18, PBS buffer and antibiotics, respectively. **(B)** Determination of the MIC and MBC values of *Ca*IFNi-18 against a spectrum of bacterial pathogens. **(C)** Inhibition curves of *Ca*IFNi-18 at MIC-fold concentrations across eight bacterial strains. **(D–E)** Cytotoxicity of *Ca*IFNi-18 in CAK and RAW 264.7 cells, as determined by a CCK-8 assay. **(F)** Hemolytic activity of *Ca*IFNi-18 toward MRBCs. (C–F) All the data are presented as the mean ± SD from one representative experiment (*n* = 3 independent samples). The statistical significance of the inhibition curves in (C) was evaluated using two-way ANOVA. Significant differences (*p* < 0.05) are denoted by different letters. For visual clarity in (C), significance is marked exclusively at the final time points.

The antibacterial efficacy of *Ca*IFNi-18 was initially determined by a disc diffusion assay. As clearly shown in Fig 7A, discs impregnated with *Ca*IFNi-18 exhibited distinct inhibition zones against all eight bacterial strains, in stark contrast with the negative control, in which no inhibitory zone was observed, revealing its wide antimicrobial spectrum. To further assess its antibacterial potency, the MIC and MBC values for *Ca*IFNi-18 against the tested bacterial strains were measured. As summarized in Fig 7B, *Ca*IFNi-18 demonstrated potent antibacterial activity against both G^-^ and G^+^ bacteria, with MIC values ranging from 9.2 to 74.0 μg/mL and MBC values ranging from 18.5 to 148.0 μg/mL. Notably, the growth of two G^-^ (*V. harveyi* and *V. parahaemolyticus*) and two G^+^ bacteria (*S. agalactiae* and *S. iniae*) was markedly suppressed under sub-MIC concentrations of *Ca*IFNi-18 (Fig 7C).

Next, we evaluated the cytotoxicity of *Ca*IFNi-18 toward RAW 264.7 and CAK cells. As shown in Fig 7D, the derived peptide *Ca*IFNi-18 had a negligible effect on the viability of mammalian cells. Even at concentrations as high as 128 μM, the viability remained 86.25% in CAK cells and 92.00% in RAW 264.7 cells. Similarly, at a concentration of 128 μM, *Ca*IFNi-18 exhibited slight hemolytic activity (3.58%) toward MRBCs (Fig 7E). In summary, *Ca*IFNi-18 possesses potent broad-spectrum antibacterial efficacy and exceptional biocompatibility *in vitro*.

### *Ca*IFNi-18 exerts bactericidal activity through inducing membrane depolarization and disrupting membrane integrity, accompanied by an *in vitro* DNA-binding capacity

**Fig 8:**
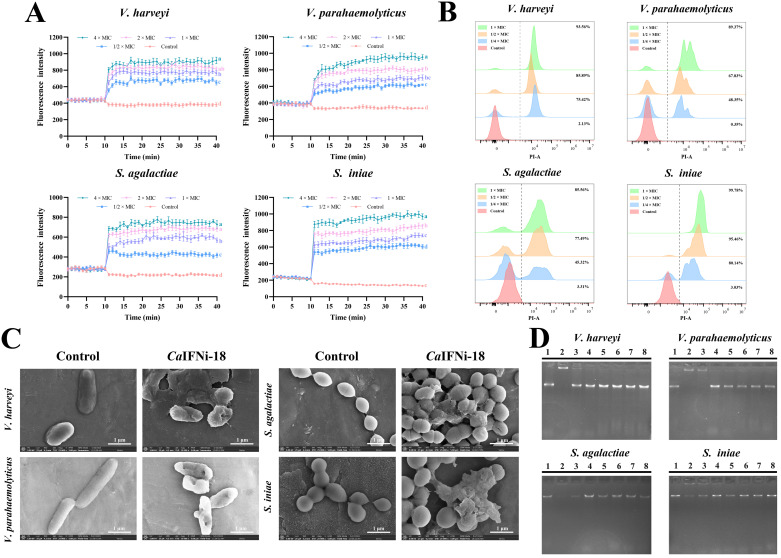
*Ca*IFNi-18 mediates its bactericidal effect by inducing membrane depolarization, disrupting membrane integrity, and binding to genomic DNA. **(A)** Detection of bacterial membrane depolarization induced by *Ca*IFNi-18 via DiSC_3_-5 staining. **(B)** Changes in membrane permeability induced by *Ca*IFNi-18 through flow cytometric analysis. The percentage of PI-positive cells is shown in the upper right corner. **(C)** Alteration of bacterial morphology after treatment with 5 × MIC *Ca*IFNi-18 for 1 h via SEM observation. The scale bar represents 1 μm. **(D)** Binding activity of *Ca*IFNi-18 to bacterial genomic DNA. The bacterial DNA was mixed with different concentrations of peptide at 37 °C for 30 min. Lane 1: 32 μM BSA; Lanes 2–8: 32, 16, 8, 4, 2, 1, 0.5 μM *Ca*IFNi-18. Data are presented as the mean ± SD from one representative experiment (*n* = 3 independent samples). Statistical significance for (A) was evaluated using two-way ANOVA. Significant differences (*p* < 0.05) are denoted by different letters. For visual clarity in (A), significance is marked exclusively at the final time points.

To elucidate the antibacterial mechanisms of *Ca*IFNi-18, we first employed the voltage-sensitive probe DiSC_3_-5 to assess its ability to elicit bacterial membrane depolarization. As shown in Fig 8A, compared with the control, the addition of *Ca*IFNi-18 to bacterial cultures in 96-well plates triggered a dose-dependent increase in fluorescence intensity, indicating its ability to induce bacterial membrane depolarization. A PI uptake assay was subsequently performed to determine the effects of *Ca*IFNi-18 on the membrane integrity of these pathogens. As shown in Fig 8B, *Ca*IFNi-18 caused substantial disruption of bacterial membrane integrity, facilitating the penetration of PI dye into the cytoplasm, and the effects on membrane permeabilization were concentration dependent. In addition, the effect of *Ca*IFNi-18 on membrane integrity was verified by SEM. The control bacteria maintained an intact cellular architecture with smooth surfaces, whereas those treated with *Ca*IFNi-18 displayed visible ultrastructural alterations, such as pore formation, shrinkage with cytoplasmic extrusion and cellular lysis (Fig 8C). Afterward, we investigated whether *Ca*IFNi-18 was able to interact with bacterial genomic DNA after entry into the cytoplasm. The results revealed a concentration-dependent binding ability of *Ca*IFNi-18 to both G^-^ and G^+^ bacterial DNA *in vitro* (Fig 8D). These findings suggest that *Ca*IFNi-18 exerts its antibacterial function primarily via alterations in membrane potential, integrity and ultrastructure coupled with *in vitro* DNA binding.

### *Ca*IFNi-18 has efficient therapeutic effects on bacterial infection *in vivo*

**Fig 9:**
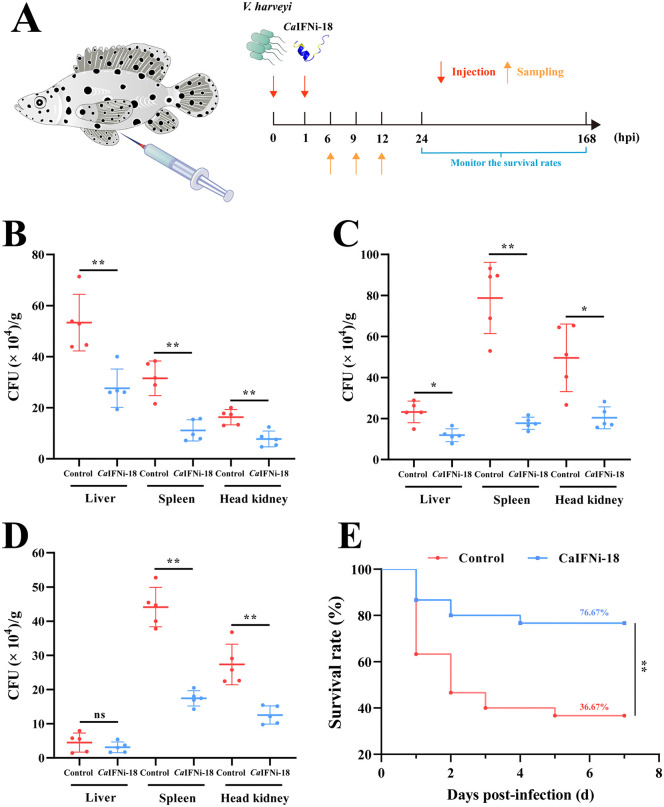
*Ca*IFNi-18 exhibits considerable therapeutic efficacy *in vivo* following infection with *V. harveyi.* **(A)** Schematic diagram of the *in vivo* experimental design. **(B–D)** The groupers were injected i.p. with 5 × 10^4^ CFU of *V. harveyi* per fish and subsequently injected with *Ca*IFNi-18 at 1 hpi. The bacterial burden in the immune tissues was quantified at 6 (B), 9 (C), and 12 h (D) post challenge. **(E)** Therapeutic efficacy of *Ca*IFNi-18 on survival rates among groupers following bacterial challenge. (B–D) Data are presented as the mean ± SD from one representative experiment (*n* = 5 biological replicates). (E) Thirty fish were used per treatment group (*n* = 30). Statistical significance was evaluated using the Mann‒Whitney U test for (B–D) and the log-rank (Mantel‒Cox) test for (E). *p < 0.05; **p < 0.01; ns: not significant.

To evaluate the therapeutic efficacy of *Ca*IFNi-18 *in vivo*, groupers were challenged via i.p. injection with 100 μL of *V. harveyi*, followed by the administration of *Ca*IFNi-18 at 1 hpi (Fig 9A). Initially, we measured the bacterial loads in the liver, spleen and head kidney at 6 h (Fig 9B), 9 (Fig 9C) and 12 hpi (Fig 9D). Strikingly, compared with those in the immune tissues of the control groupers, the bacterial loads in the immune tissues of the groupers treated with *Ca*IFNi-18 were much lower. Moreover, we monitored the survival rates over one week and observed that compared with the control treatment, *Ca*IFNi-18 effectively increased the survival rate by 40% (Fig 9E). Together, these results demonstrate that *Ca*IFNi-18 has potent *in vivo* therapeutic efficacy against *V. harveyi* infection.

### *Ca*IFNi-18 is a critical domain for the antibacterial bioactivity of *Ca*IFNi

**Fig 10:**
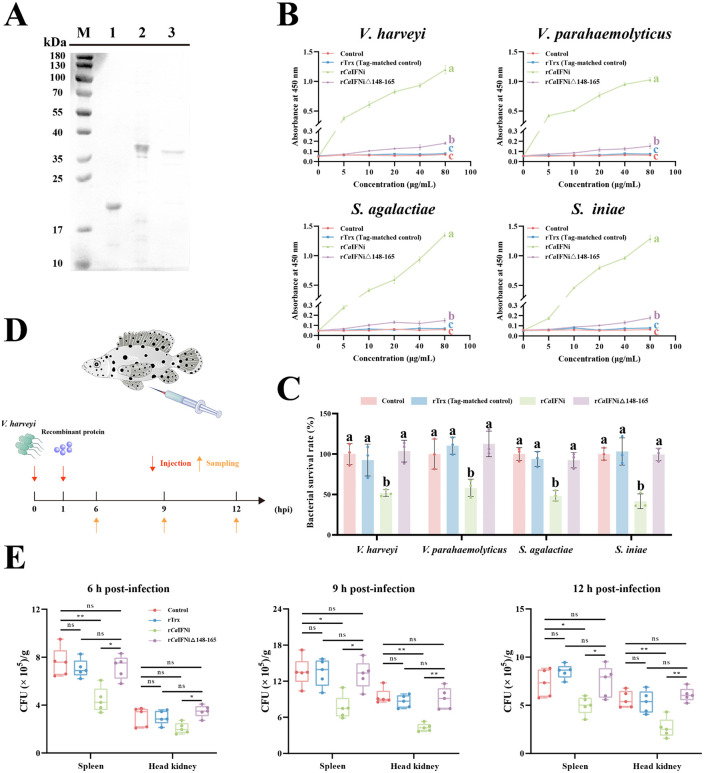
The *Ca*IFNi-18 region is indispensable for the antibacterial activity of *Ca*IFNi both *in vivo* and *in vitro.* **(A)** Analysis of recombinant *Ca*IFNi△148-165 protein purification from *E. coli* by SDS‒PAGE. Lanes 1 to 3 represent the purified rTrx, r*Ca*IFNi and r*Ca*IFNi△148-165 proteins, respectively. **(B)** ELISA of r*Ca*IFNi△148-165 binding to various aquatic bacterial pathogens. **(C)** Antibacterial effect of r*Ca*IFNi△148-165 against bacterial pathogens determined by plate counting. **(D)** Schematic diagram of the *in vivo* experimental design. **(E)** The groupers were injected i.p. with 5 × 10^4^ CFU of *V. harveyi* per fish and subsequently injected with recombinant protein (rTrx, r*Ca*IFNi or r*Ca*IFNi△148-165) or PBS at 1 hpi. The bacterial burden in the immune tissues was quantified at 6, 9, and 12 h post challenge. PBS and rTrx treatments served as the control and tag-matched control, respectively. All the data are presented as the mean ± SD from one representative experiment (*n* = 3 independent samples for B-C; *n* = 5 biological replicates for E). Statistical significance was evaluated using two-way ANOVA for (B), one-way ANOVA for (C) and the Mann‒Whitney U test for (E). Significant differences (*p* < 0.05) are denoted by different letters in (B–C). For visual clarity in (B), significance is marked exclusively at the highest concentration. *p < 0.05; **p < 0.01; ns: not significant.

To further investigate whether the antimicrobial activity of the *Ca*IFNi-18 peptide is functionally relevant within the full-length *Ca*IFNi protein, a truncation mutant devoid of this segment, designated *Ca*IFNiΔ148-165, was successfully constructed, expressed, and purified (Fig 10A). As shown in Fig 10B, an ELISA revealed that compared with wild-type r*Ca*IFNi, r*Ca*IFNiΔ148-165 almost completely lost the ability to bind to bacterial pathogens. Similarly, in contrast with r*Ca*IFNi, the truncation abolished the antibacterial efficacy of r*Ca*IFNiΔ148-165 against *V. harveyi*, *V. parahaemolyticus*, *S. agalactiae*, and *S. iniae* (Fig 10C). Furthermore, we explored the antibacterial activity of r*Ca*IFNi and r*Ca*IFNiΔ148-165 *in vivo* using a grouper *V. harveyi* infection model (Fig 10D). In line with the *in vitro* results, compared with the control or r*Ca*IFNiΔ148-165 treatments, rCaIFNi significantly potentiated bacterial clearance in tissues, whereas no significant difference in bacterial load was observed between the r*Ca*IFNiΔ148-165 group and the control group (Fig 10E). Collectively, these results suggest that the *Ca*IFNi-18 region is critical for the antibacterial effects of *Ca*IFNi, suggesting its potential role as the functional core of the full-length protein.

### Deletion of the *Ca*IFNi-18 segment impairs the interaction of *Ca*IFNi with bacterial membranes

**Fig 11:**
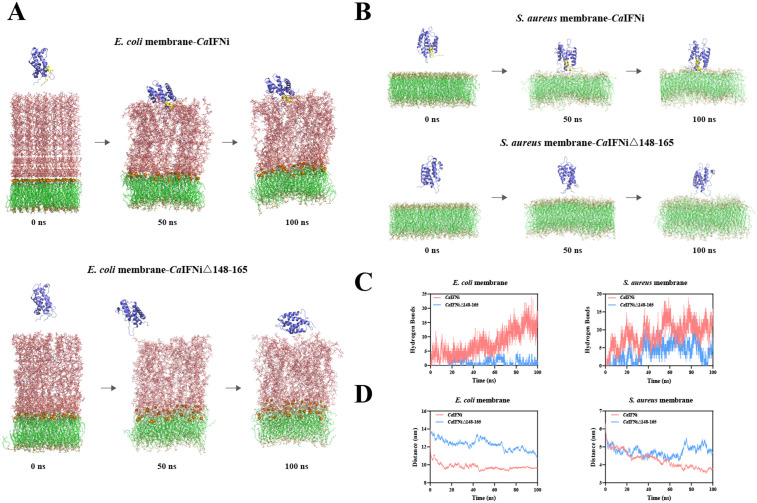
Deletion of the *Ca*IFNi-18 region attenuated the interaction between *Ca*IFNi and bacterial membranes in MD simulations. **(A-B)** MD simulation snapshots of the interaction between *Ca*IFNi or *Ca*IFNi△148-165 and the G^-^ outer membrane (A) or G^+^ membrane (B) at 0, 50, and 100 ns. Within the full-length *Ca*IFNi, the *Ca*IFNi-18 region is colored yellow. LPS is shown on the top leaflet of the G^-^ outer membrane, whereas the phosphorus atoms of lipid A are shown as tan spheres. **(C)** Hydrogen bonds of protein‒membrane interaction systems during MD simulations. **(D)** Distance between the COM of *Ca*IFNi or *Ca*IFNi△148-165 and the bacterial membrane.

To further determine the structural basis for the membrane binding capacity of *Ca*IFNi and the functional significance of the *Ca*IFNi-18 region, all-atom MD simulations were conducted using full-length *Ca*IFNi and the truncation mutant *Ca*IFNiΔ148-165. As illustrated in Fig 11A and 11B, full-length *Ca*IFNi successfully approached both G^-^ and G^+^ bacterial membranes, with its C-terminal region clearly embedding into the membrane system. Conversely, *Ca*IFNiΔ148-165 exhibited a markedly reduced interaction with the membrane and failed to establish a stable insertion throughout the 100 ns simulation. Notably, this impairment was evidenced by distinctly fewer hydrogen bonds (Fig 11C) and a consistently greater COM distance from the bacterial membranes for *Ca*IFNiΔ148-165 than for the full-length protein (Fig 11D). Collectively, our computational results demonstrate that *Ca*IFNi-18 is the critical region mediating the binding and anchoring of *Ca*IFNi to bacterial membranes.

## Discussion

As central mediators of innate immunity, type I IFNs rapidly induce the expression of hundreds of interferon-stimulated genes, resulting in cell-intrinsic antiviral states [4,20,21]. This antiviral function of type I IFNs is highly conserved across all vertebrates [22,23]. During bacterial infections, however, type I IFNs play disparate roles in host immunity, which hinges upon many elements, such as the site of the infection, virulence factors, and replication mechanisms [24]. Notably, mammalian (human and mouse) IFN-βs have been reported to possess limited bactericidal capacity in acidic environments, whereas strongly cationic type I IFNs in other jawed vertebrates exhibit broad-spectrum antimicrobial potency [9,25]. Among teleosts, both Group I and Group II IFNs exhibit classical antiviral functions. For instance, IFNc, IFNd and IFNh from *Lateolabrax japonicus* have potent antiviral activities against RGNNV [26]. Similarly, IFNi from *L. crocea* acts as an antiviral cytokine against SGIV, whereas IFNb from *Mylopharyngodon piceus* has strong antiviral activity against GCRV and SVCV [17,27]. In contrast to their extensive antiviral properties, only grass carp gcIFNφ1 (gcIFNa) and Chinese sturgeon AsIFNf have broad-spectrum antibacterial functions *in vitro* [9,10]. The IFNi subgroup, a novel member of the complex type I IFN system in teleosts, has the unprecedented characteristic of three conserved disulfide bonds, with antiviral roles reported only in *L. crocea* thus far [5]. Nevertheless, its antibacterial functions remain unexplored. In this study, for the first time, we identified an interferon gene (*Ca*IFNi) from *C*. *altivelis*, which is a Group II type I IFN, and revealed its antimicrobial activity both *in vitro* and *in vivo*. Importantly, a cationic peptide derived from *Ca*IFNi, designated *Ca*IFNi-18, was identified and synthesized. We further examined its interaction with the bacterial membrane, antimicrobial activity, biosafety, and antibacterial mechanisms and ultimately evaluated its therapeutic efficacy. In addition, through the construction of the truncation mutant r*Ca*IFNiΔ148-165, we further explored the functional link between the *Ca*IFNi-18 peptide sequence and the full-length *Ca*IFNi protein.

Phylogenetic analysis revealed that *Ca*IFNi belongs to Group II IFNs, which cluster with *Lc*IFNi, *Ar*IFNc, *Sc*IFNc, *Sm*IFN1, *Po*IFN3 and *Sp*IFNc to form a distinct clade designated the IFNi group. Notably, structural modeling indicated that *Ca*IFNi possesses three unique pairs of disulfide bonds, distinguishing it from canonical Group I and Group II IFNs, which contain one and two pairs, respectively. The distinct structural architecture of *Ca*IFNi aligns with that recently described for the IFNi of *L. crocea* [5]. Furthermore, following bacterial challenge, *CaIFNi* expression increased significantly in the immune tissues (liver, head kidney and spleen) of *C. altivelis*, indicating its crucial role in antibacterial immunity. Although primarily triggered by viruses, type I IFNs can also be elicited by the majority of bacteria [28]. In rock bream, after being stimulated with LPS or *E. tarda*, the transcript levels of two *IFNd* genes were significantly upregulated in the head kidney and blood [29]. Pereiro et al. reported that the expression of turbot *IFN2*, a Group I IFN, was markedly elevated in the head kidney following exposure to *A. salmonicida* [30]. To further validate its role in antibacterial infection, the overexpression and knockdown of *Ca*IFNi were carried out prior to bacterial challenge. Our results revealed that *Ca*IFNi overexpression markedly decreased bacterial loads within immune tissues, whereas CaIFNi silencing led to a substantial increase, underscoring its essential role in antibacterial infection *in vivo*. Similarly, IFNφ1 from zebrafish and IFN1 from grass carp provide robust protection against *S. iniae* and *A. hydrophila* infections in zebrafish, respectively [9,31]. In addition to their anti-infection activity *in vivo*, some type I IFNs with strong positive charges across jawed vertebrates have direct antibacterial functions *in vitro* [9,10,25]. Intriguingly, in the current study, while *Ca*IFNi has a net charge of − 0.3, its recombinant protein r*Ca*IFNi could still bind to bacteria and exhibit potent antimicrobial effects on both G^-^ and G^+^ bacteria. A universal mechanism among antimicrobial cytokines or peptides is their initial electrostatic attraction to lipid layers, facilitated by their characteristic high positive charges [32,33]. Therefore, on the basis of the analysis of the electrostatic surface, we speculated that the antibacterial activity of *Ca*IFNi was principally attributed to its positively charged C-terminal domain.

On this basis, we synthesized *Ca*IFNi-18, a short peptide representing the C-terminal functional motif predicted to be released naturally by proteolysis and have potent antimicrobial activity. Here, we observed that *Ca*IFNi-18 was an α-helical cationic peptide according to the results of structural modeling and CD spectroscopy, in line with the characteristics of AMPs. As the most abundant and well-studied class of antimicrobial peptides, α-helical AMPs rely on their α-helix conformation to mediate their lipid interactions with the bacterial membrane and subsequent permeation [34]. Given that the primary target of AMPs is the bacterial membrane, we first utilized MD simulations to evaluate the interaction between *Ca*IFNi-18 and bacterial membranes. As a versatile computational approach, MD simulations enable in-depth exploration of peptide‒membrane interactions, revealing mechanistic insights into antimicrobial peptide functionality [35]. During the simulation processes, *Ca*IFNi-18 progressively approached both the G^+^ and G^-^ bacterial membrane systems, followed by insertion into them, which was consistent with a steady increase in the number of hydrogen bonds and a gradual reduction in the COM distance. Interestingly, despite retaining >30% helical content in CD spectroscopy, the α-helix of *Ca*IFNi-18 underwent unfolding and transitioned to a random coil conformation during the initial simulation phase. This discrepancy suggests differential conformational stability under experimental versus simulated conditions—a phenomenon similarly observed in the lead peptide sC18_4b_ [36]. Furthermore, we performed ITC assays to clarify the interaction between *Ca*IFNi-18 and bacterial membrane components. LPS, a core structural component of G^-^ bacterial outer membranes, functions as a primary biophysical barrier against antimicrobial agents and represents a strategic target for the development of antimicrobial compounds [37]. LTA, a membrane-anchored amphiphilic polymer ubiquitous in G^+^ bacteria, facilitates the adherence of cationic AMPs to bacterial surfaces with anionic phosphate moieties [38]. In this study, ITC analysis revealed high-affinity binding between *Ca*IFNi-18 and LPS (K_d_ = 137 nM) or LTA (K_d_ = 461 nM). This observation aligns with previous findings that gcIFN-20 (derived from the fifth helical region of gcIFN1) strongly interacts with LPS (K_d_ = 13 nM) [39], whereas intestinalin (P30), a derivative from the LysC N-terminal region, can directly bind to LTA through hydrophobic interactions [40]. Collectively, these findings indicate that *Ca*IFNi-18 may exert its potential antimicrobial function by targeting LTA in G^+^ bacteria or LPS in G^-^ bacteria and in turn interacting with bacterial membranes.

To date, only a limited subset of type I IFN derivatives in fish, such as grass carp gcIFN-20, zebrafish AMP-Z1, AMP-Z2 and Chinese sturgeon *As*IFNf-α4, have been experimentally confirmed to exhibit wide-spectrum antibacterial functions *in vitro* [6,9,25,39]. Similarly, as a novel AMP, *Ca*IFNi-18 also demonstrated potent broad-spectrum antimicrobial activity against diverse G^-^ and G^+^ bacterial strains. Nevertheless, the clinical translation of AMPs remains hindered by their potential cytotoxicity [41]; as exemplified by arenicins, Hecate-βCG and melittin, these peptides inflict significant cytotoxicity on host cells despite their extraordinary antimicrobial efficacy [42–44]. Thus, we evaluated the biosafety of *Ca*IFNi-18 and reported that *Ca*IFNi-18 exhibited negligible cytotoxicity and low hemolytic activity at the tested concentrations, suggesting its extraordinary biocompatibility.

Cationic AMPs employ diverse modes of action against bacterial pathogens, principally by exerting bactericidal effects via membrane disruption following initial electrostatic adsorption [45]. Given this, we initially assessed its effects on the bacterial cell membrane to further elucidate the antibacterial mechanisms of *Ca*IFNi-18 *in vitro*. The results revealed that *Ca*IFNi-18 provoked bacterial membrane depolarization and altered membrane permeabilization and cellular ultrastructure in both G^-^ and G^+^ bacteria, which is consistent with the effects of certain AMPs, such as Larimicin_78–102_ [14] and Sp-LECin [46]. In addition to membrane disruption, some AMPs penetrate bacterial membranes via transient pore formation or receptor-mediated transport, subsequently binding to intracellular targets such as nucleic acids and metabolic enzymes to induce bactericidal effects [47,48]. In line with this, we also observed that *Ca*IFNi-18 can bind to bacterial DNA *in vitro*. Therefore, we concluded that *Ca*IFNi-18 may exert its antibacterial effects principally by disrupting bacterial membrane integrity and binding to bacterial DNA. As a typical cationic α-helical AMP, *Ca*IFNi-18 resembles classical AMPs such as LL-37 and magainins in that it binds electrostatically to negatively charged bacterial membranes to facilitate insertion and disruption [49]. Nevertheless, unlike certain potent AMPs, such as arenicins and melittin, whose application is often hindered by severe host cell cytotoxicity, *Ca*IFNi-18 is highly specific for membrane targeting, achieving broad-spectrum efficacy and excellent biocompatibility [50].

Despite their potent bactericidal activity *in vitro*, most AMPs fail to achieve the desired therapeutic efficacy *in vivo* owing to their physiological complexity [51]. Here, we further evaluated the therapeutic efficacy of *Ca*IFNi-18 against *V. harveyi* infection *in vivo*. *V. harveyi*, a G^-^ marine pathogen, has recently been identified as the primary causative agent of vibriosis outbreaks in grouper aquaculture [52,53]. In this study, injection with *Ca*IFNi-18 significantly reduced bacterial loads in grouper immune tissues and increased survival rates by 40% compared with those in the control group after one week, indicating its great potential as a therapeutic candidate for antibacterial applications. Although its efficacy *in vitro* was relatively modest compared with that of potent AMPs, the strong *in vivo* protection provided by *Ca*IFNi-18 strongly implies that *Ca*IFNi has additional immunomodulatory effects that are similar to those of other IFN-derived peptides, such as LPS neutralization [39]. Consistent with our results, CXCL20a and hepcidin have been shown to confer therapeutic efficacy in fish against *A. hydrophila* infection [54,55].

Although the *Ca*IFNi-18 peptide demonstrates exceptional *in vivo* and *in vitro* antimicrobial activity and biosafety, whether it truly represents the critical functional region of the full-length *Ca*IFNi protein remains unclear. Therefore, the truncation mutant r*Ca*IFNiΔ148-165, which lacks the *Ca*IFNi-18 segment, was constructed. Encouragingly, the deletion of this peptide domain completely abrogated the protein’s ability to bind to and kill bacteria *in vitro* as well as its therapeutic efficacy against bacterial infection *in vivo*, indicating that the *Ca*IFNi-18 region is likely a critical functional domain conferring direct bactericidal properties to *Ca*IFNi. Furthermore, MD simulations revealed that the deletion of the *Ca*IFNi-18 segment attenuated the interaction between the protein and bacterial membranes, as evidenced by decreased hydrogen bonding and increased COM distance. These *in silico* findings strongly corroborate our experimental results, indicating that *Ca*IFNi-18 is critical for membrane interaction. Similarly, in Chinese sturgeon, while the type I IFN *As*IFNf protein and its derivative *As*IFNf-α4 peptide possessed antimicrobial activity and membrane-disrupting capacity, deletion of the α4 domain abolished these functions in the *As*IFNf-Δα4 protein [10].

In summary, we identified and characterized a Group II type I IFN gene, *Ca*IFNi, in *C*. *altivelis* with a distinctive triple-disulfide bond architecture. Through *in vivo* and *in vitro* assays, we first revealed the antibacterial potency of the new subgroup of IFNi from fish type I IFNs. Moreover, a novel α-helical cationic AMP, *Ca*IFNi-18, derived from the C-terminal domain of *Ca*IFNi, was identified and found to have membrane-targeting ability, broad-spectrum antibacterial activity against both G^-^ and G^+^ bacteria *in vitro* and extraordinary biocompatibility. Further mechanistic investigations revealed that the peptides first target LPS or LTA of the bacterial membrane and then interact with and disrupt them, potentially followed by entry into the cytoplasm and binding to genomic DNA. Notably, *Ca*IFNi-18 has potent therapeutic efficacy against *V. harveyi* infection *in vivo*. More importantly, using a truncation mutant (r*Ca*IFNiΔ148-165), we found that *Ca*IFNi-18 constituted the critical bactericidal domain of the full-length protein, as its deletion not only abolished both antibacterial activity *in vitro* and therapeutic efficacy *in vivo* but also critically impaired membrane-targeting ability, as revealed by MD simulations. This study demonstrates for the first time the direct antibacterial activity of teleost IFNi and defines its derived peptide, *Ca*IFNi-18, as a pivotal region mediating this function, elucidating the antimicrobial activity and mechanism of the peptide. These findings expand the functional repertoire of type I IFNs and their derivatives and identify highly promising candidate molecules for antimicrobial drug development in aquaculture and beyond.

## Materials and methods

### Ethics statement

All the healthy *C. altivelis* used in this study were purchased from a mariculture farm situated in Wenchang city (Hainan, China). Prior to experimentation, the groupers were maintained in a recirculating sea water system for at least one week for acclimation. To exclude potential interference from preexisting bacterial infections, five fish were randomly selected for aseptic tissue sampling followed by spread-plate culture analysis as previously described [56]. All the experiments involving animals were in strict compliance with the protocols approved by the animal research ethics committee of Hainan University (HNUAUCC-2024-00140).

### Pathogenic bacteria and cell lines

*Edwardsiella tarda* (ETA1), *V. alginolyticus* (HN08155), *V. harveyi* (QT520), and *Streptococcus agalactiae* (LFY-5) were isolated from diseased marine fish in Hainan Province and preserved in our laboratory. *S. iniae* (29177), *V. parahaemolyticus* (ATCC17802), *Escherichia coli* (ATCC8739) and *Staphylococcus aureus* (ATCC6538) were purchased from the China General Microbiological Culture Collection Center. Among them, *V. harveyi* is resistant to ampicillin, while *E. tarda* and *S. agalactiae* are kanamycin resistant. The optimized conditions for culturing diverse pathogenic bacteria are as follows: *S. agalactiae* and *S. iniae* in BHI medium at 30 °C; *V. alginolyticus*, *V. harveyi*, *V. parahaemolyticus* and *E. tarda* in LB medium at 30 °C; and *E. coli* and *S. aureus* in LB medium at 37 °C. All the bacteria were cultivated to the mid-exponential phase prior to the initiation of formal experiments.

This research utilized two types of cell lines: mouse macrophages (RAW 264.7) and *C. altivelis* head kidney cells (CAK). RAW 264.7 cells, maintained in our laboratory, were cultivated in DMEM (Gibco, USA) supplemented with 10% fetal bovine serum (FBS) at 37 °C with 5% CO_2_. CAK cells, previously established by our laboratory [57], were grown in L-15 medium (Gibco, USA) supplemented with 15% FBS at 26 °C.

### Molecular cloning and identification of *Ca*IFNi

The open reading frame (ORF) for the IFNi gene from *C. altivelis*, named *Ca*IFNi, was obtained from our transcriptome library and amplified from cDNA of the spleen tissues with the specific primers *Ca*IFNi-F and *Ca*IFNi-R (S1 Table). The purified DNA products were subsequently ligated into the pEASY-T1 vector (TransGen, China) and subsequently transformed into *Escherichia coli* DH5α competent cells, among which positive clones were screened for sequencing.

The prediction for the three-dimensional (3D) structure model of *Ca*IFNi was conducted via AlphaFold3. PyMOL (version 2.6, Schrödinger, LLC) was used to visualize the net charge distribution and the predicted 3D structure. The ExPASy server was used to assess the physical and chemical parameters of the *Ca*IFNi protein. Additionally, multiple sequence alignment between *Ca*IFNi and other homologs was carried out by using the ESPript 3.0 server with a *Ca*IFNi 3D structure file. Phylogenetic tree analysis was performed with the neighbor-joining algorithm in MEGA 9.0 software.

### Expression analysis of *Ca*IFNi in humpback grouper under normal conditions and after *V. harveyi* stimulation

To analyze the tissue distribution of *Ca*IFNi, a variety of tissues (blood, liver, skin, muscle, intestine, heart, gill, spleen, brain, stomach and head kidney) were dissected from 15 pathogen-free humpback groupers, with five identical tissues pooled into one composite sample. The samples were subsequently stored in RNAstore reagent (Tiangen, China) for subsequent RNA extraction and cDNA synthesis. The mRNA expression level of *CaIFNi* was measured by quantitative reverse transcription–PCR (qRT‒PCR) using the housekeeping gene *RPL13* as an internal reference [58]. The qRT‒PCR primer sequences are listed in S1 Table, and the relative expression level was analyzed via the 2^-ΔΔCt^ method.

To investigate the modulation of *Ca*IFNi expression following *V. harveyi* infection, *C. altivelis* was divided into two groups, with each group containing 15 individuals. In the control group, *C. altivelis* received an intraperitoneal (i.p.) injection of 100 μL of PBS, whereas the challenged group was injected i.p. with an equal volume of *V. harveyi* suspension (2 × 10^6^ CFU/mL). Immune tissues (spleen, liver and head kidney) were collected at 6, 12, 24, and 48 h postinjection (hpi), preserved in RNAstore reagent and homogenized as composite samples (five identical tissues per sample).

### Effects of *Ca*IFNi overexpression and knockdown on bacterial resistance *in vivo*

To overexpress *Ca*IFNi *in vivo*, the coding sequence of *Ca*IFNi was cloned and inserted into the *Eco*R V site of the pCN3 vector, yielding the recombinant plasmid p*Ca*IFNi. Extraction of the endotoxin-free plasmid p*Ca*IFNi was performed with an EndoFree Plasmid Kit (Tiangen, China). *C. altivelis* with an average weight of 14.6 ± 1.8 g were separated into three groups, and each group (*n* = 20) received intramuscular injections of 100 μL of PBS (set as the control group), pCN3 (200 μg/mL) or p*Ca*IFNi (200 μg/mL). The expression levels of *Ca*IFNi in the head kidney and spleen were measured at 5 d post-injection (dpi) via qRT‒PCR. Moreover, all the groups were injected i.p. with 100 μL *V. harveyi* suspension (5 × 10^5^ CFU/mL), followed by head kidney and spleen collection at 6, 9 and 12 hpi for the quantification of bacterial loads.

To achieve *in vivo* knockdown of *Ca*IFNi, the T7 RiboMAX Express RNAi System (Promega, USA) was adopted to synthesize the targeting siRNA, designated Si*Ca*IFNi, with the specific primers listed in S1 Table. Healthy *C. altivelis* (20 individuals per group) were intramuscularly administered 100 μL of PBS (control) or si*Ca*IFNi/si*Ca*IFNi-C (200 μg/mL). Afterward, five fish were euthanized to assess the knockdown efficiency via qRT‒PCR at 12 hpi, and concurrently, the remaining fish were challenged with 100 μL of a *V. harveyi* suspension (5 × 10^5^ CFU/mL). The head kidney and spleen were dissected to determine the bacterial burden at 6, 9 and 12 hpi.

### Western blot analysis

The tissue samples were lysed in RIPA buffer (Beyotime, China) supplemented with protease inhibitors (Beyotime, China). The lysates were centrifuged at 14,000 rpm for 15 min at 4 °C, and the supernatants were mixed with loading buffer and heated. The proteins were separated by 12% SDS‒PAGE and transferred onto a polyvinylidene difluoride (PVDF) membrane (Millipore, USA) using an eBlot semidry transfer system (GenScript, USA). The membrane was blocked with 5% skim milk for 1 h, washed with PBST, and incubated with primary antibodies for 1 h. The primary antibodies used were mouse anti-His (bsm-33004 M; Bioss) and mouse anti-β-actin (HC201-01; TransGen). Following three PBST washes, the membrane was incubated with the secondary antibody, HRP-goat anti-mouse IgG (bs-0296G-HRP, Bioss), for 1 h. Finally, protein levels were detected using Seven Super ECL Prime substrate (Seven, China).

### Expression and preparation of the recombinant protein *Ca*IFNi (r*Ca*IFNi) and its truncation mutant r*Ca*IFNi△148-165

The sequence encoding the mature peptide of *Ca*IFNi (excluding the signal peptide) was ligated into the prokaryotic expression plasmid pET-32a at the *Eco*R V restriction site, resulting in the expression of a recombinant protein with a 21 kDa fusion partner (rTrx) that mainly consists of a Trx-His dual-tag [59]. Afterward, the recombinant plasmid was transformed into *E. coli* BL21 (DE3) cells, and protein expression was induced using 0.5 mM IPTG at 20 °C for 20 h, after which the truncation mutant r*Ca*IFNi△148-165 was induced using 0.5 mM IPTG at 16 °C for 36 h. The induced proteins were detected by 12% SDS‒PAGE, while purification and concentration quantification were in accordance with our previously established methods [60].

### Binding ability of r*Ca*IFNi and its mutant to bacteria

The binding ability of r*Ca*IFNi and its mutant to bacteria was assessed on the basis of a modified ELISA method from a previous study [60]. Briefly, 96-well plates were coated with 100 μL of bacterial suspension (1 × 10^8^ CFU/mL) at 4 °C for 12 h. Then, the plates were rinsed three times with TBST and blocked with 5% BSA solution for 1 h. After being washed with TBST, 100 μL of r*Ca*IFNi, r*Ca*IFNi△148-165 or rTrx (tag-matched control) at various concentrations was added to the plates and incubated for 3 h at room temperature, with PBS serving as a blank control. The plates were subsequently washed with TBST three times and then incubated with anti-His tag antibody dilutions (1:2000, Bioss) for 1 h. After being washed with TBST, the plates were incubated with HRP-conjugated goat anti-mouse IgG antibody (1:5000, Bioss) for 1 h at room temperature. After the final washes, TMB substrate was added to each well, and the reactions were terminated with 2 M H₂SO₄. The absorbance of each well at 450 nm was detected via a microplate reader. This assay was performed in three independent experiments.

### Antimicrobial activity of r*Ca*IFNi and its mutant *in vitro*

In brief, *V. harveyi*, *V. parahaemolyticus*, *S. agalactiae* and *S. iniae* were allowed to grow to mid-exponential phase, after which they were harvested and diluted to a concentration of 1 × 10^4^ CFU/mL in PBS. Subsequently, 100 μL of 50 μg/mL rTrx (tag-matched control), r*Ca*IFNi, r*Ca*IFNi△148-165 or PBS (control) was cocultured with 100 µL of diluted bacterial suspension at 30 °C for 3 h. Next, the mixture was spread onto LB or BHI solid plates, followed by incubation at 30 °C for 16 h for colony counting. This assay was performed in three independent experiments.

### Peptide prediction, synthesis and structure modeling

The active antimicrobial region of CaIFNi was predicted using AMPA (https://tcoffee.crg.eu/apps/ampa/do) and CAMPR4 (https://camp.bicnirrh.res.in/) servers, while its potential for natural proteolytic release was evaluated with the ExPASy PeptideCutter tool. On the basis of these predictions, *Ca*IFNi-18 (KFALQKHYHTCFTWRHHA) with an amidated C-terminus was synthesized by the solid-phase method in GL Biochem (Shanghai, China), with its purity exceeding 95% as guaranteed by high-performance liquid chromatography, and its quality was confirmed by mass spectrometry. The 3D structure of *Ca*IFNi-18 was predicted using I-TASSER and visualized via PyMOL.

### CD spectroscopy

*Ca*IFNi-18 was dissolved in 50% (v/v) TFE, 30 mM SDS or 10 mM PBS to obtain a final concentration of 100 μM. Three types of peptide solutions were individually transferred to a quartz cuvette (10 mm path length). CD spectral analyses were performed on a J-1500 spectropolarimeter (Jasco, Japan) to monitor the peptide solutions in the range of 190–250 nm. The mean residual ellipticity (θ) was calculated from the acquired CD data.

### Molecular dynamics simulations

The CHARMM-GUI server was employed to generate lipid bilayer systems, where *Ca*IFNi-18, full-length *Ca*IFNi, or the deletion mutant *Ca*IFNi△148-165 was initially positioned 2 nm above the membrane interface [61]. All-atom systems have been constructed to represent peptides or proteins that interact with G^-^ bacterial outer membranes or G^+^ bacterial membranes [62,63]. To achieve charge neutralization, 150 mM NaCl was added to the systems, after which the calcium ions stabilized the LPS within the outer leaflet of the G^-^ bacterial membranes. The system parameters are documented in S3 Table. Adopting the CHARMM36 force field and TIP3P water model in GROMACS 2021.2, all-atom MD simulations were executed for 100 ns with a 2 fs time step [64,65]. The CHARMM-GUI standard NVT/NPT protocol with a temperature of 310 K was utilized for the preceding equilibration. Molecular visualization was performed using Visual Molecular Dynamics (VMD) v1.9.4. To evaluate the conformational dynamics of *Ca*IFNi-18, *Ca*IFNi and *Ca*IFNi△148-165 in membrane systems, the root mean square deviation (RMSD), radius of gyration (Rg), center-of-mass (COM) distance and number of hydrogen bonds were analyzed from the resulting trajectories.

### Isothermal titration calorimetry (ITC)

ITC assays were performed using a MicroCal PEAQ-ITC instrument (Malvern, USA) at 25 °C. LPS (Solarbio, China), LTA (Sigma, USA) and *Ca*IFNi-18 were dissolved in 10 mM HEPES buffer (pH 7.0). In a typical titration, 13 injections of 500 μM *Ca*IFNi-18 were added to the reaction cell containing either 25 μM LPS or 50 μM LTA at 180 s intervals, with a stirring speed of 750 rpm. The heat of *Ca*IFNi-18 dilution in buffer was subtracted from the raw data prior to integration in MicroCal Origin 5.0 software, after which the corrected data were analyzed using one-site ﬁtting models.

### Disc diffusion assay

To preliminarily determine the antibacterial activity of *Ca*IFNi-18, a disc diffusion assay was conducted with reference to a previous method [48]. In brief, the bacterial cells were harvested, diluted in PBS to a concentration of 1 × 10^7^ CFU/mL and subsequently spread onto agar plates. After that, blank paper disks were placed onto the plates, after which 20 μL of PBS (negative control), 1 mg/mL ampicillin/kanamycin (positive control) or 1 mg/mL *Ca*IFNi-18 was added to the discs dropwise, after which the plates were incubated for 16 h at either 37 °C or 30 °C. Finally, zones of inhibition were documented utilizing a GenoSens 2100 gel imaging system (Clinx, China).

### MIC and MBC assays

The MIC and MBC of *Ca*IFNi-18 were evaluated in accordance with a twofold dilution method [66]. Briefly, bacterial suspensions were prepared in the respective liquid culture media (LB broth for gram-negative strains and BHI broth for gram-positive strains) at a concentration of 2 × 10^6^ CFU/mL. One hundred μL of bacterial suspension was mixed with equal volumes of *Ca*IFNi-18 serial dilutions in 96-well plates, resulting in final concentrations ranging from 1 to 128 μM, whereas PBS and ampicillin/kanamycin served as the negative and positive controls, respectively. After incubation for 16 h, the absorbance of each well at 600 nm was measured using a microplate reader. The MIC was defined as the minimum concentration of *Ca*IFNi-18 at which bacterial growth was completely suppressed. To determine the MBC of *Ca*IFNi-18, 100 μL of the mixture was spread onto agar plates, followed by incubation for 16 h. The MBC was defined as the lowest concentration at which no colonies formed on the plates. This assay was performed in three independent experiments.

### Inhibition curve assay

Various concentrations (100 μL) of the peptide solutions at the MIC were cultured with equal volumes of bacterial suspension (2 × 10^6^ CFU/mL) in 96-well plates. Afterward, the absorbance of each well at 600 nm was detected every hour by a microplate reader for 9 consecutive hours. This assay was performed in three independent experiments.

### Cytotoxicity and hemolytic activity assays

The cytotoxicity of *Ca*IFNi-18 was evaluated using cell lines (RAW 264.7 and CAK) with reference to a previous method with some modifications [67]. The cells were inoculated into 96-well plates (1 × 10^4^ cells/well) and cultivated overnight. Subsequently, 10 μL of serial dilutions of *Ca*IFNi-18 were added to the cells to achieve a final concentration of 1–128 μM, followed by coculture for 24 h. Then, 10 μL of CCK-8 solution (Biosharp, China) was added to each well. After incubation for 2 h, the absorbance at 450 nm was measured by a microplate reader. The medium alone and containing cells functioned as the blank and positive control, respectively. The cell viability was calculated as (OD_treatment_ − OD_blank_)/(OD_control_ − OD_blank_) × 100%. This assay was performed in three independent experiments.

Mouse red blood cells (MRBCs) were utilized to assess the hemolytic activity of *Ca*IFNi-18 as previously described [68]. Briefly, MRBC were isolated, harvested by centrifugation at 800 × g for 10 min, washed three times with 0.9% NaCl solution, and subsequently prepared as a 4% (v/v) suspension in 0.9% NaCl solution. Afterward, 100 μL MRBC suspension was mixed with 100 μL serial dilutions of *Ca*IFNi-18 (the final concentration ranged from 1 to 128 μM) and incubated at 37 °C for 1 h. After centrifugation, the supernatant was collected, and the absorbance was measured at 540 nm. 0.9% NaCl and 1% Triton X-100 with MRBC served as the negative and positive controls, respectively. The hemolysis rate = (OD_treatment_ – OD_negative_)/(OD_positive_ − OD_negative_) × 100%. This assay was performed in three independent experiments.

### Membrane depolarization assay

3,3’-Dipropylthiadicarbocyanine iodide (DiSC_3_-5), a cationic dye, was used to detect bacterial membrane depolarization levels, and the assay was conducted as previously described [48]. Briefly, bacteria were washed three times with HEPES buffer (5 mM HEPES, 20 mM glucose), and a bacterial suspension (OD_600_ = 0.05) was prepared either in buffer A for G^-^ bacteria (5 mM HEPES, 20 mM glucose, 100 mM KCl and 2 mM EDTA) or in buffer B (5 mM HEPES, 20 mM glucose and 100 mM KCl) for G^+^ bacteria. Afterward, a 100 μL aliquot of the bacterial suspension was transferred to a black 96-well plate, and 1 μM DiSC3-5 was added. After incubation for 90 min, the fluorescence intensity (excitation λ = 622 nm; emission λ = 670 nm) of each well was measured by a microplate reader for 10 min. Subsequently, 50 μL of the MIC-fold *Ca*IFNi-18 solution was added to the mixture, and the fluorescence intensity was monitored at one-minute intervals for 30 min, while deionized water functioned as a negative control. This assay was performed in three independent experiments.

### Propidium iodide (PI) uptake assay

The effects of *Ca*IFNi-18 on bacterial membrane permeability were estimated via a PI uptake analysis performed with flow cytometry. In brief, the bacterial suspension was adjusted to a concentration of 1 × 10^8^ CFU/mL in PBS, followed by incubation with MIC-fold *Ca*IFNi-18 for 1 h. Afterward, PI was added to the mixture at a final concentration of 10 μg/mL and incubated at room temperature for 30 min. Finally, the samples were analyzed by flow cytometry (Beckman, USA), and the data were processed with FlowJo software (v.10.9).

### Scanning electron microscopy (SEM)

To investigate the morphological effects of *Ca*IFNi-18 on bacterial cells, a modified SEM protocol was adopted [69]. A bacterial suspension (1 × 10⁸ CFU/mL) in 0.9% NaCl solution was treated with 5 × MIC *Ca*IFNi-18 or 0.9% NaCl (control) for 1 h. Following incubation, the cells were rinsed three times with 0.9% NaCl and then fixed in 2.5% glutaraldehyde at 4 °C overnight. After fixation, the cells were pelleted, washed and subsequently dehydrated in a graded ethanol series (30%, 50%, 70%, 90%, and 100% absolute ethanol) for 15 min each. The dehydrated samples were lyophilized in a vacuum freeze dryer overnight, coated with gold and then observed by SEM (Verios G4 UC, Thermo Scientific, USA).

### DNA binding assay

This assay was performed to evaluate the DNA binding ability of *Ca*IFNi-18 to bacterial genomic DNA according to a previous method [70]. Briefly, bacterial genomic DNA was extracted using a TIANamp Bacteria DNA Kit (Tiangen, China). Afterward, the purified bacterial DNA (100 ng) was treated with *Ca*IFNi-18 at concentrations ranging from 0.5 to 32 μM, while 32 μM BSA-treated DNA was used as a control. After a 30-minute incubation at 37 °C, the samples were analyzed by 1.2% agarose gel electrophoresis and visualized using a gel documentation system.

### Therapeutic efficacy of *Ca*IFNi-18 against *V. harveyi* infection *in vivo*

To determine whether *Ca*IFNi-18 had therapeutic effects on *V. harveyi*, a grouper model of *V. harveyi* infection was established by administering 100 μL of bacterial suspension at a concentration of 5 × 10^5^ CFU/mL. At 1 hpi, *C. altivelis* (12.7 ± 3.0 g) were randomly assigned to one of two groups (*n* = 50). One group was injected i.p. with 100 μL of PBS, whereas the other group received an injection of 100 μL of *Ca*IFNi-18 (100 μg/mL). Afterward, the liver, spleen and head kidney were collected at 6, 9, and 12 hpi to quantify the bacterial load, and the mortality rate of the fish was monitored daily for one week.

### Antibacterial activity of recombinant *Ca*IFNi and its mutant against *V. harveyi in vivo*

To assess the antibacterial activity of the recombinant protein r*Ca*IFNi and its truncation mutant *in vivo*, groupers (16.9 ± 2.7 g) were challenged via i.p. injection with 5 × 10^4^ CFU of *V. harveyi*. At 1 hpi, the fish were randomly assigned to four groups (*n* = 20) and administered a 100 μL injection of PBS (control) or the respective recombinant proteins (rTrx as a tag-matched control, r*Ca*IFNi, or r*Ca*IFNi△148-165) at a concentration of 100 μg/mL. Tissue samples from the spleen and head kidney were collected at 6, 9, and 12 hpi to measure bacterial loads.

### Statistical analysis

Statistical analyses were performed using GraphPad v.8.0 software. The data are presented as the means ± SDs. The significance of differences between two independent groups was determined by an unpaired Student’s t test. For multiple comparisons, one-way analysis of variance (ANOVA) was used for the plate counting assays, whereas two-way ANOVA followed by Tukey’s multiple comparisons test was used to evaluate the ELISA, inhibition curve, and membrane depolarization assay results. Survival rates were evaluated by the log-rank (Mante-Cox) test. Quantification of bacterial loads was performed via a nonparametric Mann‒Whitney U test. The significant differences are presented as *p < 0.05 and **p < 0.01.

## Supporting information

S1 Table Primers used in this study. (DOCX)

S2 Table Amino acid sequence of type I IFNs in teleosts involved in analysis of phylogenetic tree. (DOCX)

S3 Table Parameters for constructing membrane-peptide and membrane-protein systems in molecular dynamics. (DOCX)

S1 Text Rationale for the selection of bacterial surface components. (DOCX)

S1 Raw Images Raw images of Figs 3D, 4A, 8D and 10A. (PDF)
