# Correction: Noncanonical bactericidal activity of teleost type I interferon is conferred by a membrane-targeting C-terminal peptide

**DOI:** 10.1371/journal.ppat.1014570

**Published:** 2026-09-03

**Authors:** Han Zhang, Ying Wu, Xiaoyuan Zheng, Ziyu Wang, Chen Zhang, Zhenjie Cao, Jingqun Ao, Yongcan Zhou, Yun Sun

There are errors in the author affiliations. The correct affiliations are as follows:

Han Zhang¹, Ying Wu¹, Xiaoyuan Zheng¹, Ziyu Wang¹, Chen Zhang¹, Zhenjie Cao¹, Jingqun Ao^2^, Yongcan Zhou^1, 3, 4^ Yun Sun^1, 3, 4^

**1** Sanya Institute of Breeding and Multiplication, School of Marine Biology and Fisheries, Collaborative Innovation Center of Marine Science and Technology, Hainan University, Hainan, China, **2** College of Marine Sciences, College of Life Sciences, Fujian Agriculture and Forestry University, Fuzhou, China, **3** Engineering Research Center of Hainan Province for Blue Carbon and Coastal Wetland Conservation and Restoration, Hainan University, Hainan, China, **4** International Joint Research Center of Hainan Province for Blue Carbon and Coastal Wetland, Hainan University, Hainan, China
