# Supplementary material for: Noncanonical bactericidal activity of teleost type I interferon is conferred by a membrane-targeting C-terminal peptide
Source: PLoS Pathog. 2026 Jul 28;22(7):e1014419. doi: 10.1371/journal.ppat.1014419 (PMC13411935; doi:10.1371/journal.ppat.1014419)
Supplement: S1 Table — (DOCX) [file ppat.1014419.s001.docx]

**S1 Table. Primers used in this study.**

| Primer | Primer sequence（5’ → 3’） | Application |
| --- | --- | --- |
| CaIFNi-F | gatatcgccaccATGACAGTGTCCTCAGTCCTGC | Gene cloning and construction of eukaryotic expression vector |
| CaIFNi-R | gatatcGTGGGCGTGGTGTCTCCA |  |
| CaIFNi-F1 | gatatcATGCCGACCTGTAAACTGGA | Construction of prokaryotic expression vector |
| CaIFNi-R1 | gatatcGTGGGCGTGGTGTCTCCA |  |
| CaIFNi-F2 | cgcggatccATGCCGACCTGTAAACTGGA | Construction of trunction mutant |
| CaIFNi-R2 | ccggaattcGTGCAGGACCCTGAGCAGA |  |
| CaIFNi-F3 | CCAGAAACTCGCTAAGTTCCA | qRT-PCR |
| CaIFNi-R3 | TCTCCACAGAGCCATCCATC |  |
| RPL13-F | CGGACGTGGCTTCACTCTG | qRT-PCR |
| RPL13-R | CTTGATGGGCATGACTGGAC |  |
| SiCaIFNi-p1 | GGATCCTAATACGACTCACTATAGCGTTAACGGTTCAGAGGT | Synthesizing  siCaIFNi |
| SiCaIFNi-p2 | AAACCTCTGAACCGTTAACGCTATAGTGAGTCGTATTAGGATCC |  |
| SiCaIFNi-p3 | GGATCCTAATACGACTCACTATAACCTCTGAACCGTTAACGC |  |
| SiCaIFNi-p4 | AAGCGTTAACGGTTCAGAGGTTATAGTGAGTCGTATTAGGATCC |  |
| SiCaIFNi-p1-C | GGATCCTAATACGACTCACTATAGCAAGCTGACCCTGAAGTT | Synthesizing  siCaIFNi-C |
| SiCaIFNi-p2-C | AAAACTTCAGGGTCAGCTTGCTATAGTGAGTCGTATTAGGATCC |  |
| SiCaIFNi-p3-C | GGATCCTAATACGACTCACTATAAACTTCAGGGTCAGCTTGC |  |
| SiCaIFNi-p4-C | TATAGTGAGTCGTATTAGGATCCGTGGTGGTGGTGGTGGTG |  |
