# Supplementary material for: Noncanonical bactericidal activity of teleost type I interferon is conferred by a membrane-targeting C-terminal peptide
Source: PLoS Pathog. 2026 Jul 28;22(7):e1014419. doi: 10.1371/journal.ppat.1014419 (PMC13411935; doi:10.1371/journal.ppat.1014419)
Supplement: S2 Table — (DOCX) [file ppat.1014419.s002.docx]

**S2 Table. Amino acid sequence of type I IFNs in teleosts involved in analysis of phylogenetic tree.**

| Gene | Species | Accession |
| --- | --- | --- |
| ArIFNc | *Argyrosomus regius* | AVD96636.1 |
| ArIFNd | *Argyrosomus regius* | AVD96637.1 |
| ArIFNh | *Argyrosomus regius* | AVD96638.1 |
| CiIFNa | *Ctenopharyngodon idella* | ABC87312.1 |
| CiIFNc1 | *Ctenopharyngodon idella* | AMT92190.1 |
| CiIFNc2 | *Ctenopharyngodon idella* | AMT92191.1 |
| CiIFNd | *Ctenopharyngodon idella* | AMT92192.1 |
| DrIFNa | *Danio rerio* | AAM95448.1 |
| DrIFNc1 | *Danio rerio* | NP_001104552.1 |
| DrIFNc2 | *Danio rerio* | NP_001104553.1 |
| DrIFNd | *Danio rerio* | NP_001155212.1 |
| OmIFNc1 | *Oncorhynchus mykiss* | CCV17402.1 |
| OmIFNc2 | *Oncorhynchus mykiss* | CCV17403.1 |
| OmIFNc3 | *Oncorhynchus mykiss* | CCV17404.1 |
| OmIFNc4 | *Oncorhynchus mykiss* | CCV17405.1 |
| OmIFNb1 | *Oncorhynchus mykiss* | NP_001153974.1 |
| OmIFNb2 | *Oncorhynchus mykiss* | NP_001158515.1 |
| OmIFNb3 | *Oncorhynchus mykiss* | CCV17399.1 |
| OmIFNb4 | *Oncorhynchus mykiss* | CCV17400.1 |
| OmIFNd1 | *Oncorhynchus mykiss* | CAV07949.1 |
| OmIFNe1 | *Oncorhynchus mykiss* | CCV17406.1 |
| OmIFNe2 | *Oncorhynchus mykiss* | CCV17407.1 |
| OmIFNe3 | *Oncorhynchus mykiss* | CCV17408.1 |
| OmIFNe4 | *Oncorhynchus mykiss* | CCV17409.1 |
| OmIFNe5 | *Oncorhynchus mykiss* | CCV17410.1 |
| OmIFNe6 | *Oncorhynchus mykiss* | CCV17411.1 |
| OmIFNa1 | *Oncorhynchus mykiss* | CAM28541.1 |
| OmIFNa2 | *Oncorhynchus mykiss* | NP_001153977.1 |
| OmIFNa3 | *Oncorhynchus mykiss* | CCV17397.1 |
| OmIFNa4 | *Oncorhynchus mykiss* | CCV17398.1 |
| OmIFNf1 | *Oncorhynchus mykiss* | CCV17413.1 |
| OmIFNf2 | *Oncorhynchus mykiss* | CCV17414.1 |
| PoIFN3 | *Paralichthys olivaceus* | BBA46271.1 |
| PoIFN4 | *Paralichthys olivaceus* | BBA46272.1 |
| SsIFNc1 | *Salmo salar* | ACE75692.1 |
| SsIFNc2 | *Salmo salar* | XP_014048249.2 |
| SsIFNc3 | *Salmo salar* | ACE75688.1 |
| SsIFNb1 | *Salmo salar* | ACE75691.1 |
| SsIFNb2 | *Salmo salar* | ACE75693.1 |
| SsIFNb3 | *Salmo salar* | ACE75689.1 |
| Ss IFNd | *Salmo salar* | DAA64377.1 |
| SsIFNa1 | *Salmo salar* | ABD39320.1 |
| SsIFNa3 | *Salmo salar* | ACE75687.1 |
| SsIFNa2 | *Salmo salar* | ACE75687.2 |
| SsIFNf1 | *Salmo salar* | ACE75687.3 |
| SsIFNf2 | *Salmo salar* | ACE75687.4 |
| SmIFN1 | *Scophthalmus maximus* | AID59461.1 |
| SmIFN2 | *Scophthalmus maximus* | AID59462.1 |
| SpIFNc | *Stegastes partitus* | XP_008298153.1 |
| LcIFNd | *Larimichthys crocea* | API68651.1 |
| LcIFNh | *Larimichthys crocea* | API68650.1 |
| LcIFNi | *Larimichthys crocea* | AYP67465.1 |
| CcIFNa | *Cyprinus carpio* | ADI81047.1 |
| ScIFNc | *Siniperca chuatsi* | AVJ47959.1 |
| ScIFNd | *Siniperca chuatsi* | AVJ47960.1 |
| ScIFNh | *Siniperca chuatsi* | AVJ47961.1 |
| ArIFNf | *Acipenser ruthenus* | XP_033912947.2 |
| SaIFNf1 | *Salvelinus alpinus* | XP_023991983.1 |
| SaIFNf2 | *Salvelinus alpinus* | XP_023867236.1 |
| SnIFNf | *Salvelinus namaycush* | XP_038836766.1 |
| CaIFNi | *Cromileptes altivelis* | PV927265 |
