# Supplementary material for: Noncanonical bactericidal activity of teleost type I interferon is conferred by a membrane-targeting C-terminal peptide
Source: PLoS Pathog. 2026 Jul 28;22(7):e1014419. doi: 10.1371/journal.ppat.1014419 (PMC13411935; doi:10.1371/journal.ppat.1014419)
Supplement: S3 Table — (DOCX) [file ppat.1014419.s003.docx]

**S3 Table. Parameters for constructing membrane-peptide and membrane-protein systems in molecular dynamics.**

| **Peptides/Proteins** | **Membrane** | **Box dimension (nm)** | **Atoms** |  |
| --- | --- | --- | --- | --- |
| *Ca*IFNi-18 | Outer membranes of G- bacteria (outer: 35 ECLIPA; inner: 75 PPPE, 20 PVPG, 5 PVCL2) | 8.04 × 8.04 × 20.15 | 137342 |  |
|  |  |  |  |  |
|  |  |  |  |  |
| *Ca*IFNi-18 | Membranes of G+ bacteria (POPG:TLCL2 = 4:1) | 11.42 × 11.42 × 7.24 | 96652 |  |
|  |  |  |  |  |
|  |  |  |  |  |
| *Ca*IFNi | Outer membranes of G- bacteria (outer: 35 ECLIPA; inner: 75 PPPE, 20 PVPG, 5 PVCL2) | 8.14 × 8.14 × 32.78 | 25602 |  |
|  |  |  |  |  |
|  |  |  |  |  |
| *Ca*IFNi | Membranes of G+ bacteria (POPG:TLCL2 = 4:1) | 11.10 × 11.10 × 18.96 | 20782 |  |
|  |  |  |  |  |
|  |  |  |  |  |
| *Ca*IFNi△148-165 | Outer membranes of G- bacteria (outer: 35 ECLIPA; inner: 75 PPPE, 20 PVPG, 5 PVCL2) | 8.14 × 8.14 × 32.78 | 25439 |  |
|  |  |  |  |  |
|  |  |  |  |  |
| *Ca*IFNi△148-165 | Membranes of G+ bacteria (POPG:TLCL2 = 4:1) | 11.10 × 11.10 × 18.96 | 20619 |  |
|  |  |  |  |  |
|  |  |  |  |  |
