# Supplementary material for: Noncanonical bactericidal activity of teleost type I interferon is conferred by a membrane-targeting C-terminal peptide
Source: PLoS Pathog. 2026 Jul 28;22(7):e1014419. doi: 10.1371/journal.ppat.1014419 (PMC13411935; doi:10.1371/journal.ppat.1014419)
Supplement: S1 Text — (DOCX) [file ppat.1014419.s004.docx]

**S1 Text**

**Rationale for the selection of bacterial surface components.**

The initial stage of action for cationic AMPs is fundamentally driven by electrostatic interactions with the bacterial surface. Given the substantial structural and compositional divergence between G^+^ and G^-^ bacterial envelopes, we strategically selected representative components (LPS and LTA) for our *in vitro* binding assays to accurately reflect these physiological targets.

**Lipopolysaccharide (LPS) for G^-^** **bacterial models:**

The cell envelope of G^-^ bacteria is characterized by an asymmetric outer membrane, where the outer leaflet is predominantly composed of LPS. Due to its densely packed phosphate and carboxyl groups, LPS confers a strong negative charge to the bacterial surface. It serves as the foremost permeability barrier and the primary initial docking site for cationic AMPs.

**Lipoteichoic Acid (LTA) for G^+^ bacterial models:**

G^+^ bacteria lack an outer lipid membrane but possess a thick, porous peptidoglycan layer. This layer is heavily traversed by LTA, a polyanionic polymer intercalated via a glycolipid moiety into the underlying cytoplasmic membrane. Extending outward through the cell wall, LTA imparts a dense network of negative charges to the cell surface, acting as the functional and electrostatic equivalent of LPS in mediating the initial recruitment of cationic AMPs.
