# Supplementary figures and images for: Noncanonical bactericidal activity of teleost type I interferon is conferred by a membrane-targeting C-terminal peptide
Source: PLoS Pathog. 2026 Jul 28;22(7):e1014419. doi: 10.1371/journal.ppat.1014419 (PMC13411935; doi:10.1371/journal.ppat.1014419)

**Fig 3D**

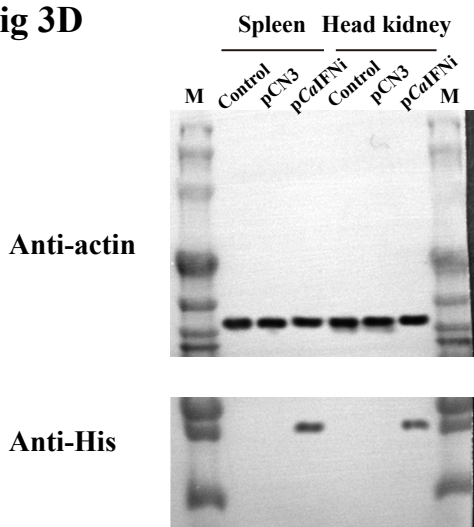

**Fig 4A**

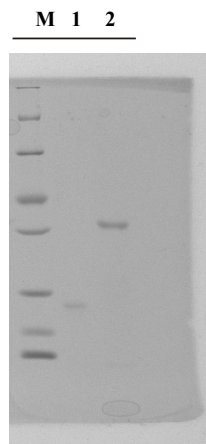

**Fig 8D**

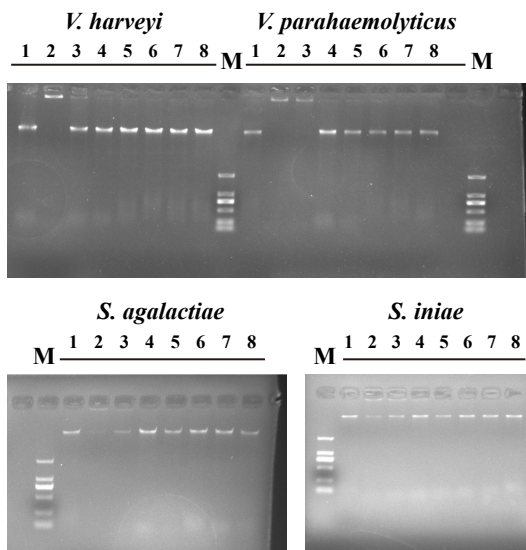

**Fig 10A**

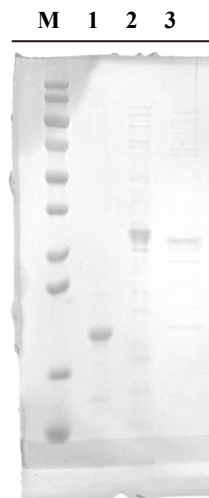

Supplement: S1 Raw Images — (PDF) [file ppat.1014419.s005.pdf]
